# An automatic method to generate domain-specific investigator networks using PubMed abstracts

**DOI:** 10.1186/1472-6947-7-17

**Published:** 2007-06-20

**Authors:** Wei Yu, Ajay Yesupriya, Anja Wulf, Junfeng Qu, Marta Gwinn, Muin J Khoury

**Affiliations:** 1 National Office of Public Health Genomics, Coordinating Center for Health Promotion, Centers for Disease Control and Prevention, Atlanta, GA, USA; 2 Department of Information Technology, Clayton State University, Atlanta, GA, USA

## Abstract

**Background:**

Collaboration among investigators has become critical to scientific research. This includes ad hoc collaboration established through personal contacts as well as formal consortia established by funding agencies. Continued growth in online resources for scientific research and communication has promoted the development of highly networked research communities. Extending these networks globally requires identifying additional investigators in a given domain, profiling their research interests, and collecting current contact information. We present a novel strategy for building investigator networks dynamically and producing detailed investigator profiles using data available in PubMed abstracts.

**Results:**

We developed a novel strategy to obtain detailed investigator information by automatically parsing the affiliation string in PubMed records. We illustrated the results by using a published literature database in human genome epidemiology (HuGE Pub Lit) as a test case. Our parsing strategy extracted country information from 92.1% of the affiliation strings in a random sample of PubMed records and in 97.0% of HuGE records, with accuracies of 94.0% and 91.0%, respectively. Institution information was parsed from 91.3% of the general PubMed records (accuracy 86.8%) and from 94.2% of HuGE PubMed records (accuracy 87.0). We demonstrated the application of our approach to dynamic creation of investigator networks by creating a prototype information system containing a large database of PubMed abstracts relevant to human genome epidemiology (HuGE Pub Lit), indexed using PubMed medical subject headings converted to Unified Medical Language System concepts. Our method was able to identify 70–90% of the investigators/collaborators in three different human genetics fields; it also successfully identified 9 of 10 genetics investigators within the PREBIC network, an existing preterm birth research network.

**Conclusion:**

We successfully created a web-based prototype capable of creating domain-specific investigator networks based on an application that accurately generates detailed investigator profiles from PubMed abstracts combined with robust standard vocabularies. This approach could be used for other biomedical fields to efficiently establish domain-specific investigator networks.

## Background

Collaboration among investigators and research groups in the biomedical field has become increasingly crucial to achieving success in the understanding of complex diseases such as cancer and heart disease [1]. As a result, many networks and consortia have been established to promote collaboration and data sharing. Networking of investigators and searching for potential collaborators in a specific research domain will be especially important in the genomics era, which provides an opportunity to apply basic research to the promotion of human health and disease prevention. The HuGENet initiative to develop a "network of investigator networks" in human genome epidemiology [2] illustrates the efforts of a diverse, global research community that is committed to accelerating the development and synthesis of knowledge on genetic variation and human diseases [3]. As more researchers recognize the importance of establishing networks to enhance efficiency and reduce redundancy in scientific research, major challenges include identifying investigators with particular interests and acquiring contact information for building new networks and updating this information for existing networks.

PubMed [4], offering access to the MEDLINE database of citations and abstracts of biomedical research articles, provides one of the most valuable information resources for tracking the progress of biomedical research through the published literature; it can also be used to find collaborators and investigators by authorship. Citation analyses that address the structure of scientific collaboration networks have been done many times [5-8]. Our approach shows how information contained in PubMed abstracts and author affiliation strings can be used to extend existing networks even further by identifying more investigators who may be new collaborators. In this paper, we present a novel PubMed-based approach to building a dynamic investigator network with detailed investigator profiles that include institutional affiliation, country of origin, email address, and publication history. We illustrate our concept using a prototypical web-based system for building an investigator network.

## Methods

### Data sources

We used 20,000 randomly selected PubMed abstracts from articles published between 2001 and 2005 (PubMed data) to determine the extent of affiliation data in PubMed. We used a continuously updated literature database of studies relevant to human genome epidemiology (HuGE Pub Lit [9]) to create a prototype web-based system for building an investigator network. As of October 19, 2006, the HuGE Pub Lit database contained 23,876 PubMed abstracts of gene-disease association studies (HuGE PubMed data).

The National Center for Biotechnical Information Entrez Programming Utilities (NCBI E-utility) [10] was used to retrieve full PubMed records containing title, authors, abstract, and affiliations based on PubMed Unique Identifier (PMID). We took advantage of the fact that most PubMed abstracts are indexed with National Library of Medicine medical subject headings (MeSH) terms by NCBI staff. We used a standard vocabulary, Unified Medical Language System (UMLS) metathesaurus (version 2006AB) [11], to index PubMed abstracts by converting MeSH terms to UMLS concept unique identifiers (CUIs). To enrich the capacity of UMLS to handle gene information, we incorporated Entrez gene records into the UMLS metathesaurus, substituting Entrez gene IDs for the UMLS CUIs. Gene symbols were indexed manually using these Entrez gene IDs [12]. The MeSH hierarchy tree [13] was used to provide "children" concepts for query terms.

### Affiliation parsing

#### PubMed affiliation string format

While building the affiliation parsing tool, we found that over 80% of the affiliation strings in PubMed articles adhered to the following format:

[address component], [address component], ..., [country]. [email].

#### Country name lookup list

We created a country lookup table containing country names and their synonyms based on International Organization for Standardization 3166 country codes [14] and UMLS. The UMLS metathesaurus lists numerous synonyms for country names, for example, United States, US, U.S.A., etc. Using this table, country names could be assigned to 86% of the affiliation strings. The remaining affiliation strings could not be parsed for one or more of the following reasons: 1) a noncountry geographic location, such as a city or state, was provided instead of a country name; 2) the affiliation was written in a language other than English; or 3) the affiliation was provided in an unconventional format. To handle the first two scenarios, we created a custom country name list by manually inspecting these affiliation strings and adding the geographic locations as synonyms for countries. For example, if "Beijing" was in an affiliation string without country information, we added "Beijing" to the lookup table as a synonym for China in the custom country name list. We used a second-run parsing algorithm if the affiliation was provided in unconventional format.

#### Email address parsing pattern

A regular expression pattern was used to find and parse the email address in the affiliation string (see detail in the appendix file)

#### Institution key work list

To capture this information, including some in languages other than English, we created an institution key word list (Table 1).

**Table 1:** Keyword list for parsing institution information

**Expression Pattern**	**Institution Name**	**Languages**	**Examples**
univ	University	English German Dutch Spanish French Italian Portuguese	University of Michigan Technische Universität München Vrije Universiteit Medical Center Hospital General Universitario Université de la Réunion Università degli Studi Universidade Federal do Rio Grande do Sul
institu	Institute	English German Spanish French Portuguese Swedish	National Institutes of Health Institut für Arbeitsphysiologie an der Universität Dortmund Instituto de Parasitología y Biomedicina 'López Neyra' Institut Pasteur Instituto Português de Oncologia Karolinska Institutet
hospital	Hospital	English Spanish Italian Portuguese	Queen's University of Belfast Hospital Ramón y Cajal Hospital Casa Sollievo della Sofferenza Hospital de Santo Espírito de Angra do Heroísmo
college	College	English French	Medical College of Georgia College de France
cent	Center	English French Italian Portuguese	Memorial Sloan-Kettering Cancer Center Centre de Médecine Préventive Centro Studi Farmaco-Tossicodipendenze Centro de Histocompatibilidade do Sul
foundat	Foundation	English	Janssen Research Foundation
school	School	English	Menzies School of Health Research
system	System	English	North Shore-Long Island Jewish Health System
acad	Academy	English Dutch	Chinese Academy of Sciences Academisch Centrum voor Tandheelkunde Amsterdam (ACTA)
facul	Facility	English Spanish French Portuguese	Istanbul Faculty of Medicine Facultad de Medicina de la UANL Faculté de médecine Xavier Bichat Faculdade de Medicina de São José do Rio Preto
labora	Laboratory	English French	Abbott Laboratories Laboratoire de Génétique Moléculaire et d'Histocompatibilité CHU Morvan
clin	Clinic	English French Italian	Mayo Clinic Clinique Marc Linquette Policlinico Borgo Roma
infirm	Infirmary	English	Royal Infirmary of Edinburgh
agenc	Agency	English	International Agency for Research on Cancer

Detailed affiliation parsing algorithm can be found in the appendix file.

#### Example of parsed affiliation

Original affiliation string: Pulmonary and Critical Care Medicine, Yale University School of Medicine, 300 Cedar Street, TAC-441S, PO Box 208057, New Haven, CT 06520, USA. geoffrey.chupp@yale.edu.

Parsed information:

Full address: Pulmonary and Critical Care Medicine, Yale University School of Medicine, 300 Cedar Street, TAC-441S, PO Box 208057, New Haven, CT 06520, USA

Country: USA (CUI code: C0041703)

Institution: Yale University School of Medicine

Email: geoffrey.chupp@yale.edu

### Web-based demonstration version of the system implementing the methodology

**Figure 1:**
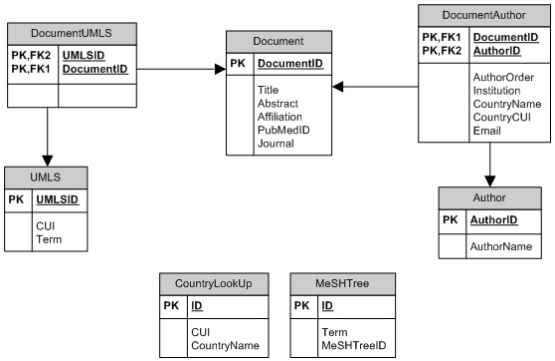
Relational database schema. Note: UMLS – Unified Medical Language System. CUI – Concept Unique Identifier. MeSH – Medical Subject Heading. PK – Primary Key. FK – Foreign Key

We generated a relational database that linked PubMed abstract content, detailed investigator profiles, and indexed UMLS/Entrez gene concepts. Because PubMed abstracts provide an affiliation only for the first author, the parsed affiliation information was linked to the first author of the corresponding publication abstract. A diagram of the database schema is shown in Figure 1.

Java J2EE 1.4 [15] was used to build the web-based system combined with the open-source frameworks Hibernate [16] and Struts [17]. The Microsoft SQL server was used as the back-end database.

### Performance Evaluation

Two test sets were used to assess the accuracy of the parsing application. We extracted all 311 records (HuGE PubMed test data) added to HuGE Pub Lit between October 20, 2006, and November 3, 2006, and randomly selected 311 articles (PubMed test data) that had been added to the PubMed database during the same period

By using preterm birth as a test case, we tested the system's ability to dynamically create domain-specific investigator networks. After consulting with an expert in the domain of preterm birth, the following query was used to search the database: "prematurity or infant, premature or infant, low birth weight or labor, premature." We compared the members of the dynamic investigator network built by using our system with the membership of an existing network, the International *PRE*term *BI*rth *C*ollaborative (PREBIC), which includes a subgroup for study of genetic factors in preterm birth [18].

To further evaluate the performance of the methodology, we invited domain experts in the fields of human genome epidemiology of preterm birth, Chlamydia infection and HIV infection to participate in the tests. The experts performed the search using the Investigator Browser by choosing their own search terms. Each expert reviewed the list of investigators generated by the Investigator Browser and labeled the ones they had collaborated with or recognized as investigators in their field; they also provided us with investigator names that they expected to find but that were not on the list. We used this information to estimate sensitivity of the methodology.

## Results

### Extent of affiliation information in general PubMed abstracts and HuGE PubMed abstracts

In our sample of general PubMed abstracts, 87% had affiliation strings; those lacking them were mostly nonresearch publications such as biographies, comments, or letters. In all, 98.6% of HuGE PubMed abstracts contained affiliation strings. Email information was available in about 40% of both general PubMed records and HuGE PubMed records. In both datasets, affiliation profiles could be constructed for about 20% of all authors (Table 2).

**Table 2:** Affiliation information available from records in PubMed and HuGE Pub Lit

	Affiliation Availability*	**Email Availability**†	**Authors with affiliation**‡	**First authors with affiliation**§
**HuGE PubMed data**	98.6%	43.0%	19.8%	98.8%
**General PubMed data**	87.3%	40.3%	22.3%	90.7%

*Affiliation availability: number of documents that have affiliation string/total number of documents.

†Email availability: number of documents that have valid email addresses/total number of documents.

‡Authors with affiliation: number of first authors with affiliation/number of all authors.

§First authors with affiliation: number of first authors with affiliation/number of first authors.

### Performance Evaluation

Our parsing tool was able to obtain all email addresses in the valid format by using regular expression pattern matching (see Methods). Performance of affiliation parsing is given in Table 3.

**Table 3:** Affiliation parsing performance

	**Country**		**Institution**	
	Parsable*	Accuracy†	Parsable	Accuracy

**General PubMed test data**	92.1%	94.0%	91.3%	86.8%
**HuGE PubMed test data**	97.0%	91%	94.2%	87.0%

*Parsable: number of abstracts that have country or institution information/number of abstracts that have affiliation information.

†Accuracy: number of abstracts that have correct country or institution information/number of abstracts that have affiliation information.

Comparing the list of investigators generated by the methodology with information provided by domain experts showed that our approach could identify about 70% – 85% of investigators in three different research areas with the selection of the first or last authors only while over 90% of investigators were identified if all authorship was considered (Table 4).

**Table 4:** Comparison of investigators identified by experts and the methodology

**Domain**	**Query**	# Investigator experts identified in the methodology-generated list (%) *		**# Investigator experts identified**	**#Investigator the methodology generated**
				
		First/Last Author only	All Author		
Preterm Birth	preterm birth or premature	40 (83.33%)	46 (95.83%)	48	502(F/L)^§ ^1694(All)
HIV	hiv	97 (83.62%)	111 (95.69%)	116	518(F/L)^§ ^1997(All)
Chlamydia trachomatis	Chlamydia trachomatis	17 (70.83%)	24 (100%)	24	19 (F/L)^§ ^68(All)

* %: the number of the investigators in the methodology-generated list/the number of investigators experts identified.

^§^F/L:First/Last Authors option in Investigator Browser; All: All Authors option in Investigator Browser.

By using a domain-specific query (see Methods) and the web-based prototype system, we dynamically built an investigator network for the HuGE field focused on genetic factors in preterm birth. The HuGE Pub Lit database contained 122 relevant abstracts, from which we identified 548 investigators (authors), including 178 who were represented as either first or last authors. Detailed profiles for each investigator included the number of publications in PubMed, number of publications in HuGE Pub Lit, and number of HuGE publications as the first or last author. Of the 10 genetics investigators within the PREBIC network, 9 were included in the list of investigators returned by web-based network building system. One investigator was missed because he had not yet published any articles that were included in HuGE Pub Lit.

### Web-based demonstration version of the system

**Figure 2:**
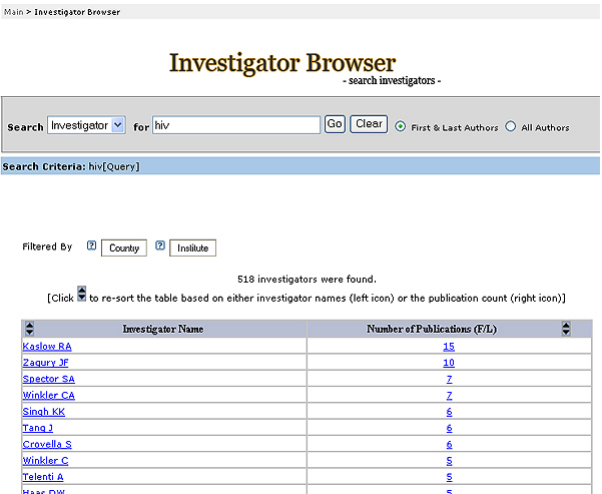
Results of Investigator Browser search for HIV investigator network in human genome epidemiology.

**Figure 3:**
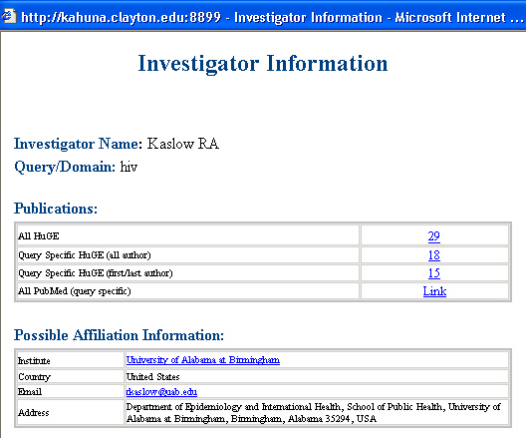
Investigator Browser showing an investigator detail profile in HIV investigator network in human genome epidemiology.

**Figure 4:**
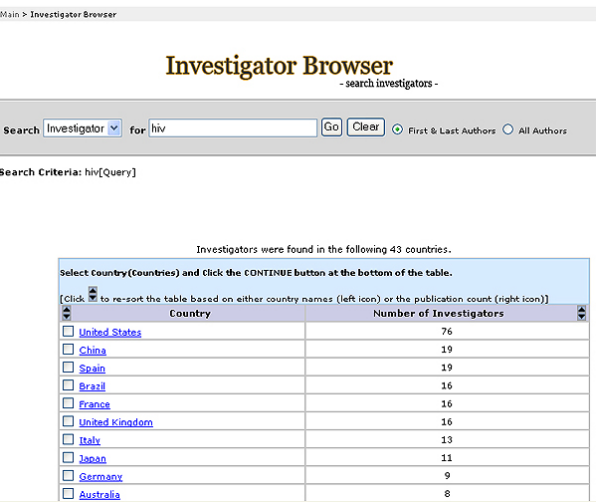
Investigator Browser presentation of country distribution in HIV investigator network in human genome epidemiology.

With this system, we were able to retrieve articles using a query for a specific domain of interest identified by indexed UMLS terms, all possible children terms, and text word searching of title and abstract to generate a dynamic, user-defined network with a list of authors and detailed author profiles. This approach allows users to construct domain-specific investigator networks (Figure 1); browse investigators and corresponding investigator profiles (Figure 2); and stratify the investigators by country (Figure 3) and institution (Figure 4).

The demonstration version of the system implementing this methodology can be accessed [25].

## Discussion

Investigator networking and collaboration is common practice in modern scientific research, aided by the emergence of new technology, especially the Internet. Collaboration can greatly enhance research by increasing the volume of high-quality data available to investigators and accelerating progress toward research goals [19,20]. The HuGENet movement [2] has made great efforts to promote global collaboration among investigators conducting population-based research in genetic epidemiology. Recently, HuGENet launched an initiative to establish a "network of networks" across the field by registering existing networks, teams, and investigators to share data, develop standards, facilitate the confirmation of research findings, and reduce duplication of effort [1,21]. Domain-specific investigator networks created by our prototype system could be instrumental in identifying additional investigators to recruit to these networks.

Citation analysis of the published literature is a reliable method for describing scientific collaboration networks by identifying and connecting authors that have made contributions in the same research field [7]. MEDLINE is the largest component of PubMed, the freely accessible online database of biomedical journal citations and abstracts created by the U.S. National Library of Medicine (NLM). With the assistance of information technology, PubMed allows for quick elucidation of comprehensive investigator networks. In addition to abstract content and author names, PubMed provides limited affiliation information (including country, institution, and contact information), which has practical value for network building. The ability to search MeSH-indexed abstracts allows domain-specific investigator networks to be generated dynamically. Quick and up-to-date answers to the "3W" questions (Who, Where, and What) can be obtained without soliciting investigators.

Affiliation strings in PubMed records have been used to analyze the geographic distribution of published studies [22,23]. However, the heterogeneity of country names has required time-consuming manual extraction procedures that precluded the generation of large datasets. We successfully developed and implemented an automated approach that uses the UMLS to accurately and robustly parse the affiliation string. Our affiliation parsing strategy demonstrates the capacity to extract investigator profile information efficiently from PubMed records.

Although our approach provides a new way to explore and build investigator networks from PubMed, it has many limitations. First, PubMed records identify authors only by last name and first initial, which can create some ambiguity in investigator networks generated by our system. However, this may not be a consideration in the future, because PubMed recently started to provide full names in XML format. Second, because PubMed provides affiliation information only for the first author, detailed investigator profiles can be generated only for investigators with publications in which they are first author. Third, indexing of institutions could not be completely automated because of inconsistency in the institution names provided by authors, a problem that could be addressed by establishing an international registry of research institutions. Finally, PubMed does not include all biomedical journals, especially those published in other countries. Adapting the current system for other data sources such as EMBASE [24] could result in more comprehensive, dynamically created investigator networks.

## Conclusion

The new approach presented in this paper uses information available in PubMed abstracts as an efficient way to identify potential collaborators in a particular research domain. We demonstrated this approach in the field of human genome epidemiology, but it could be applied to any field represented in PubMed to track investigators and dynamically create domain-specific investigator networks.

## Competing interests

The author(s) declare that they have no competing interests.

## Authors' contributions

WY developed the methodology, built the prototype web-based system, and drafted the manuscript. AY was involved in the system design and the data analysis and helped in manuscript preparation. AW participated in design of the system evaluation, data collection and analysis. JQ was involved in the system design and configuration, and data management. MG assisted with study design, provided advice on the project and revised the draft manuscript. MJK oversaw the project and revised the draft manuscript. All authors read and approved the final document.

**Figure 5:**
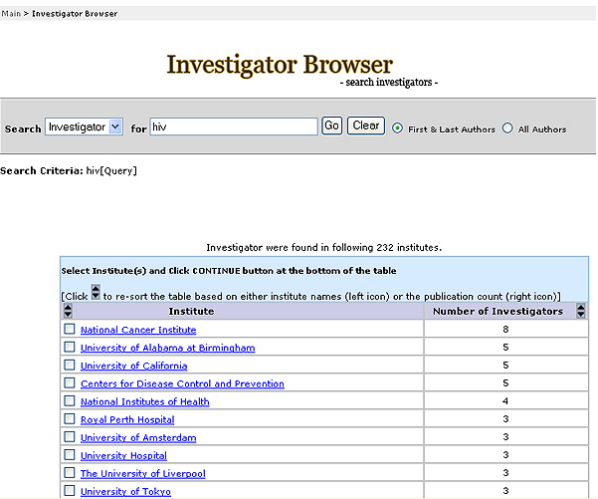
Investigator Browser presentation of institution distribution in HIV investigator network in human genome epidemiology.

## Pre-publication history

The pre-publication history for this paper can be accessed here:



## Supplementary Material

Additional File 1 Two columns: UMLS CUI, search terms corresponding to countries (CUI) Click here for file

Additional File 2 detail parsing algorithm for affiliation strings. Click here for file
