# Supplementary material for: An automatic method to generate domain-specific investigator networks using PubMed abstracts
Source: BMC Med Inform Decis Mak. 2007 Jun 20;7:17. doi: 10.1186/1472-6947-7-17 (PMC1931433; doi:10.1186/1472-6947-7-17)
Supplement: Additional File 2 — detail parsing algorithm for affiliation strings. [file 1472-6947-7-17-S2.doc]

**Regular expression for parsing email address**

[a-z0-9.\-_+]+@([a-z0-9\_]+\.)+(com|net|org|edu|int|mil|gov|arpa|biz|tr|jp|pl|tr|fr|cz|cn|au| aero|name|coop|info|pro|museum|tv|([a-z]{2}))

**Detailed steps for the affiliation parsing tool**

First Run:

1. Create an array to store all terms parsed by comma delimiters for each affiliation string;
2. Loop the array backward;
   - For the first element, get email information if the email pattern is matched;
     1. By the period delimiter, get the first element that may be a possible country string;
     2. Take the element to query the country lookup table;
     3. Get the UMLS concept unique identifier (CUI) for the given country string if found;
   - Continue with other elements by looking up the institution key word list (Table 1) and stop when a key word is matched. The string with the given matched key word is considered the institution string;
   - Put the whole affiliation string as the full address after trimming the email string if it exists.

Second Run:

For each affiliation string without country information after the first run:

1. Create an array from the customized country term list (see appendix file);
   - Loop the array by each affiliation string;
   - If the affiliation string contains one term, the corresponding country CUI will be assigned to the given affiliation.
